# HYPOTHETICAL INTERVENTIONS ON LONELINESS AND MEMORY FUNCTION AMONG US MIDDLE-AGED AND OLDER ADULTS

**DOI:** 10.1093/geroni/igae098.0528

**Published:** 2024-12-31

**Authors:** Ryo Ikesu, L Paloma Rojas-Saunero, Eleanor Hayes-Larson, Elizabeth Rose Mayeda

**Affiliations:** University of California Los Angeles, Los Angeles, California, United States; University of California Los Angeles, Los Angeles, California, United States; USC Leonard Davis School of Gerontology, Los Angeles, California, United States; University of California Los Angeles, Los Angeles, California, United States

## Abstract

Evidence has shown that middle-aged and older adults who experience persistent loneliness have lower memory function compared to those with transient loneliness or those who never feel lonely, but it remains unclear whether sustained interventions on loneliness, as opposed to a one-time intervention, are more effective in preserving late-life memory function. Researchers are increasingly using hypothetical intervention approaches to estimate the impact of population-level interventions with observational data. Using the nationally-representative Health and Retirement Study in 2006–2016 (n=16,977; median baseline age, 68), we estimated the population-level effects of two hypothetical interventions of loneliness on memory scores 8 years after baseline: [A] preventing loneliness at baseline and 4 years after baseline (sustained intervention at two time points) and [B] preventing loneliness only at baseline (one-time intervention). We used targeted maximum likelihood estimation to account for censoring and both baseline and time-varying confounding (health-related behaviors, working status, household wealth, social relationships, and health conditions). In this approach, we accounted for the possibility that people with lower memory function feel lonely more often than those with higher memory function. Compared to the natural course (i.e., no intervention), both sustained and one-time interventions were associated with slightly higher memory scores 8 years after baseline (0.026 standardized units [95% CI: 0.003–0.048] for the sustained intervention; 0.021 standardized units [95% CI: 0.005–0.037] for the one-time intervention). Our findings suggest that both sustained and one-time interventions on loneliness may preserve memory function at the population level.

